# Hyperalignment: Modeling shared information encoded in idiosyncratic cortical topographies

**DOI:** 10.7554/eLife.56601

**Published:** 2020-06-02

**Authors:** James V Haxby, J Swaroop Guntupalli, Samuel A Nastase, Ma Feilong

**Affiliations:** 1 Center for Cognitive Neuroscience, Dartmouth CollegeHanoverUnited States; 2 Vicarious AIUnion CityUnited States; 3 Princeton Neuroscience InstitutePrincetonUnited States; National Institute of Mental Health, National Institutes of HealthUnited States; Radboud UniversityNetherlands

**Keywords:** cortex, topography, population response, functional connectivity, hyperalignment, individual differences

## Abstract

Information that is shared across brains is encoded in idiosyncratic fine-scale functional topographies. Hyperalignment captures shared information by projecting pattern vectors for neural responses and connectivities into a common, high-dimensional information space, rather than by aligning topographies in a canonical anatomical space. Individual transformation matrices project information from individual anatomical spaces into the common model information space, preserving the geometry of pairwise dissimilarities between pattern vectors, and model cortical topography as mixtures of overlapping, individual-specific topographic basis functions, rather than as contiguous functional areas. The fundamental property of brain function that is preserved across brains is information content, rather than the functional properties of local features that support that content. In this Perspective, we present the conceptual framework that motivates hyperalignment, its computational underpinnings for joint modeling of a common information space and idiosyncratic cortical topographies, and discuss implications for understanding the structure of cortical functional architecture.

## Introduction

Information encoded in cortex is organized at varying spatial scales. The introduction of multivariate pattern analysis of fMRI data (MVPA; Haxby, 2012; Haxby et al., 2014; Haxby et al., 2011; Haxby et al., 2001) revealed that information can be decoded from fine-grained patterns of cortical activity (see also Bzdok, 2017; Haynes, 2015; Haynes and Rees, 2006; Hebart and Baker, 2018; Norman et al., 2006; Pereira et al., 2009; Tong and Pratte, 2012). Study of cortical functional connectivity also has revealed fine-grained topographies in the connectome (Arcaro et al., 2015; Haak et al., 2018; Heinzle et al., 2011; Jbabdi et al., 2013) that are closely related to these patterns of activity (Guntupalli et al., 2018). In the context of fMRI, fine-scale granularity in cortical patterns in fMRI data refers to voxel-by-voxel (or vertex-by-vertex) variation in response and connectivity profiles. Even finer-scale variation exists at the level of columns and neighboring neurons (Leopold et al., 2006; Park et al., 2017). The surface structure of functional cortical topographies, however, shows considerable variability across individual brains for encoding the same information (Cox and Savoy, 2003; Guntupalli et al., 2016; Haxby et al., 2011). We introduced a novel conceptual framework that models both the shared information encoded in fine-grained functional topographies and individual-specific topographic idiosyncrasies. This framework, ‘hyperalignment’ (Chen et al., 2014; Chen et al., 2015; Guntupalli et al., 2018; Guntupalli et al., 2016; Haxby et al., 2011; Xu et al., 2012), encompasses a family of computational algorithms that align shared information in a common, high-dimensional information space, rather than attempting to align functional topographies in the physical space of cortical anatomy to a canonical topography. Hyperalignment derives from the term hyperspace, to convey the paradigm shift from modeling cortical functional architecture in a three-dimensional anatomical space to modeling the information encoded in functional architecture in a high-dimensional information space. This common information space captures the ‘deep structure’ of shared information that is encoded in the variable ‘surface structure’ of idiosyncratic topographies in individual brains.

Hyperalignment algorithms construct the common information space and calculate transformations that project individually-variable patterns of neural activity and connectivity into this common model space. Information is contained in the relationships among pattern vectors – distinctions and similarities – and these relationships are preserved when the space is transformed from an individual cortical space to the common information space. In these transformations, the features that define the space in individual brains – response tuning functions and connectivity profiles – are remixed to support the same information structure with shared basis functions. Thus, in the conceptual framework of our computational model, the fundamental property of brain function that is preserved is information content, and the functional properties of local features that support that content are mutable and secondary.

Our computational framework introduces a new model of cortical functional architecture. Shared information is decomposed into overlapping basis functions that are instantiated in individual brains with idiosyncratic topographies. Cortical patterns of activity and connectivity are modeled as mixtures of these topographic basis functions. Our common model accounts for coarse areal structure insofar as the model’s shared basis set for fine-scale topographies can be mixed to identify the boundaries for functional areas in individual brains, but the topographic information embedded in the model’s shared bases is not strongly constrained by these areal boundaries, suggesting that areal structure may play a smaller role in how information is organized in cortex than was previously believed.

Here we present the fundamental concepts that underlie this approach to modeling cortical functional architecture, as well as the computational underpinnings for deriving the common information space and modeling idiosyncratic cortical topographies. We review the effectiveness and significance of these methods, their potential for increasing the sensitivity of inquiry into individual differences, and discuss the implications of this conceptual framework for understanding cortical functional architecture.

## Key concept: High-dimensional information spaces

### Vector space representation of cortical patterns

**Figure 1.**
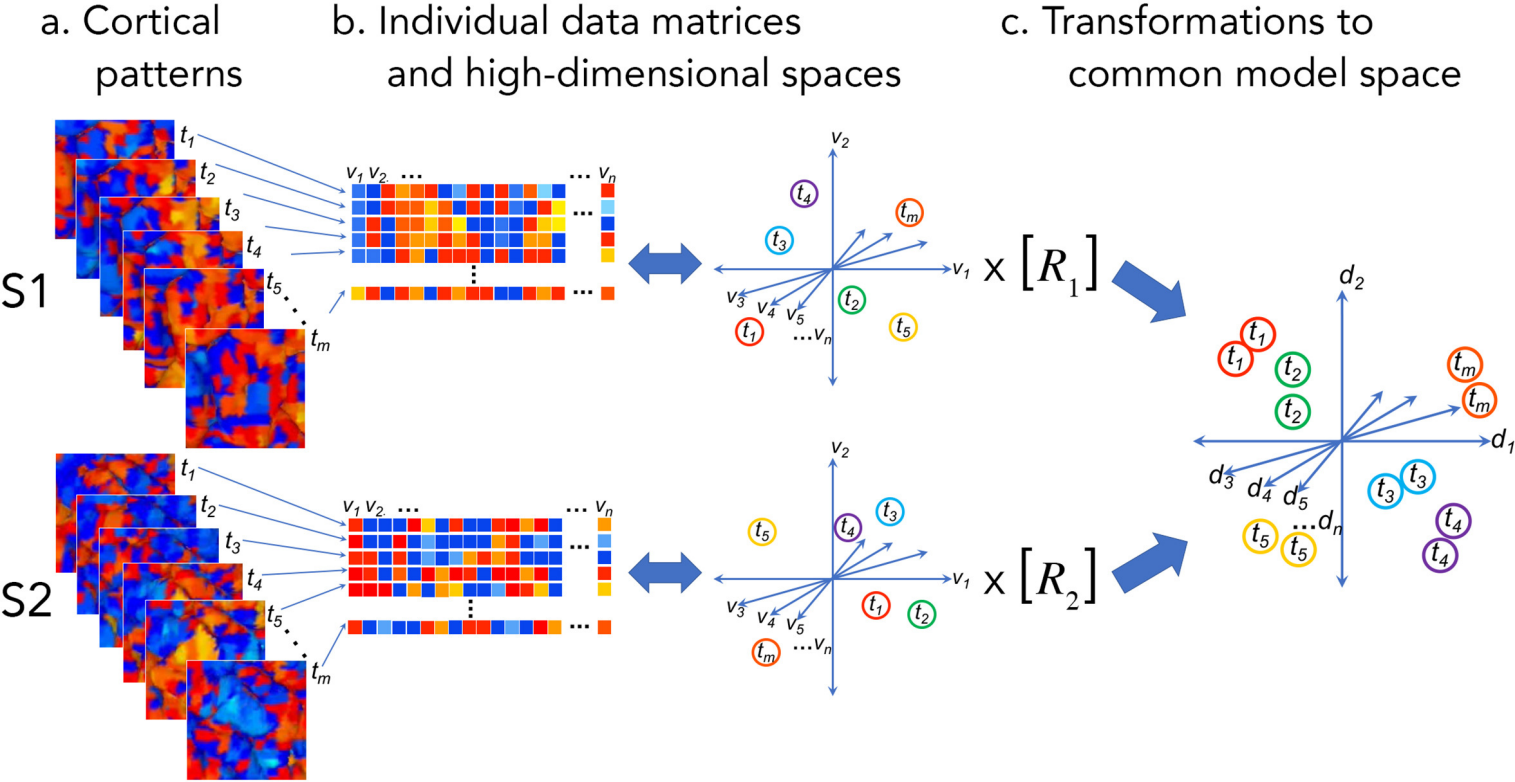
Cortical patterns as vectors in individual and common high-dimensional information spaces. Cortical functional patterns (**a**) are analyzed as data matrices of vectors in high-dimensional cortical feature spaces (**b**). Hyperalignment transforms individual information spaces based on cortical loci into a common model space in which pattern vectors for the same brain state (corresponding to a given stimulus, condition, or task) are aligned across brains (**c**). With fMRI data, cortical patterns are typically responses at different time points or connectivities to different targets (t), brain loci are voxels or cortical vertices (v), transformations are matrices (R), and common model coordinate axes are dimensions (d).

Cortical functional patterns can be represented as vectors corresponding to locations in a high-dimensional vector space where each dimension is a local measurement or element of that pattern (Figure 1a–b; Haxby et al., 2014). Instead of representing cortical function as a topographic spatial map in the three-dimensional physical space of the cortex (or a two-dimensional representation of the cortical sheet), hyperalignment models the information embedded in cortical function in this high-dimensional space (cf. Durbin and Mitchison, 1990). For fMRI data, local pattern elements are typically voxels (or vertices on the cortical surface). Patterns of neural activity measured with single-unit recording, electrocorticography (ECoG), or electro- and magnetoencephalography (EEG and MEG) can also be analyzed as pattern vectors in cortical or subcortical feature spaces in which each dimension may be a single neuron, an electrode, or a dipole, magnetic field sensor, or magnetic source. The relevance of the hyperalignment conceptual framework for modeling cortical functional architecture is not specific to any measurement modality or neural variable, and the computational procedures can also be applied to data matrices obtained with other measurement modalities (e.g. Chen et al., 2020) or even artificial neural networks (Lu et al., 2019). We will focus this perspective on human cortical activity patterns accessible with fMRI.

### Information as vector geometry

**Figure 2.**
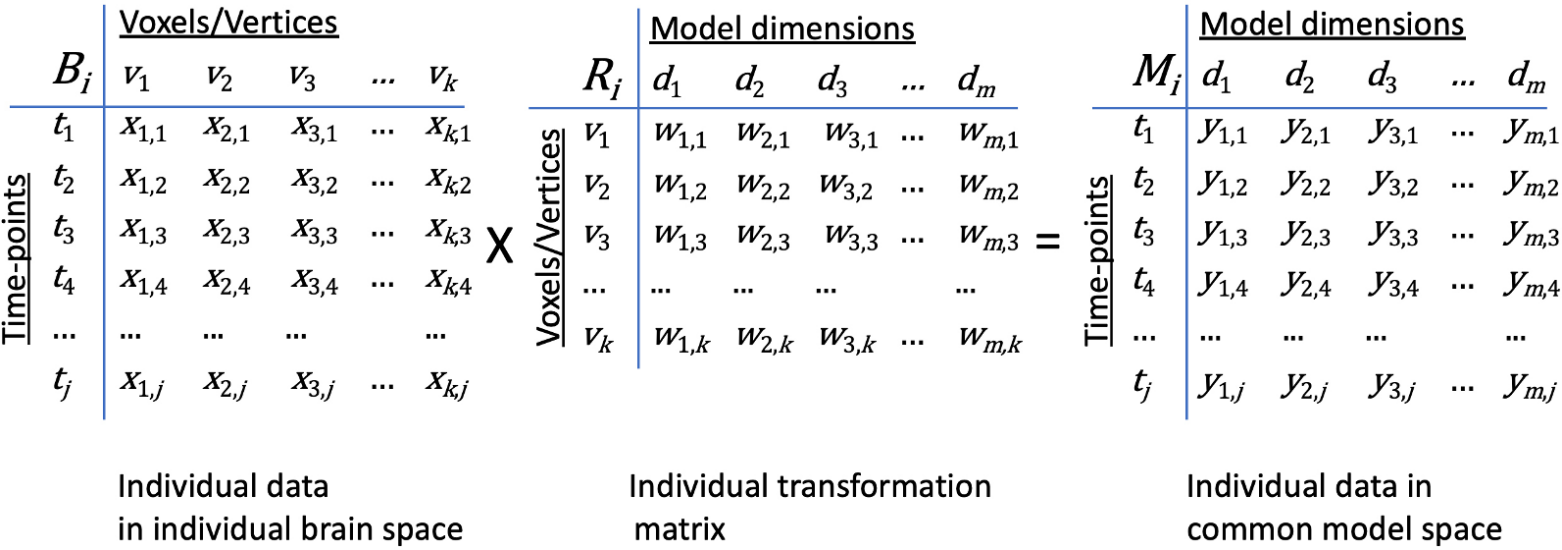
Transformation of data in individual brain coordinate space into common information space coordinates. An individual brain data matrix in cortical space (*B_i_*) consists of *j* rows of patterns (t) with *k* columns of cortical loci (v). Patterns can be neural activity for time-points, stimuli, or conditions; patterns of connectivity with targets; or other measures that vary across cortical loci. The transformation matrix for that individual (*R_i_*) consists of *k* rows of cortical loci (v) with *m* columns of model dimensions (d). Multiplying these matrices transforms the individual brain data matrix, *B_i_*, into the model space coordinates (*M_i_*) with rows of activity or connectivity patterns, *t*, in model dimension coordinates, d). Elements in brain data matrices (*x* in *B_i_*) are measures of local neural activity or connectivity of that locus with a connectivity target. Elements in transformation matrices (*w* in *R_i_*) are weights. Data in the model space matrix (*y* in *M_i_*) are weighted sums of measures from cortical loci (*x* in *B_i_*).

Matrices of brain activity and connectivity lend themselves to analysis as geometric structures in high-dimensional information spaces. The rows of these matrices are distributed cortical patterns of responses to stimuli or connectivities with cortical targets elsewhere in the brain. The columns are response or connectivity profiles for cortical loci (voxels or vertices for fMRI) in individual brains or model dimensions in the common information space (Figures 1 and 2). The row vectors may be patterns of activity at different points in time or response patterns recovered from multiple instances of a given event or experimental manipulation (using trial averaging or deconvolution) and serve as indices of the brain state corresponding to a given stimulus or cognitive state. These row vectors also may be patterns of functional connectivities between each cortical locus in a field and a locus, or connectivity target, elsewhere in the brain. Thus, the information space is populated by response pattern vectors or connectivity pattern vectors. We refer to the response or connectivity magnitudes for a given cortical location (i.e., a column in the data matrix) as a functional profile.

Here, we define ‘information’ as relationships among functional pattern vectors, typically quantified as the similarity (or dissimilarity/distance) between each pair of vectors (Connolly et al., 2012; Connolly et al., 2011; Edelman et al., 1998; Hanson et al., 2004; Kriegeskorte et al., 2008a; Kriegeskorte and Kievit, 2013; O'Toole et al., 2005). These dissimilarities index the distinctiveness of each vector relative to other vectors; vectors that are more similar to each other are located nearer to each other in vector space. This geometric framework for operationalizing information as the relationships among distributed patterns in a vector space is used in a variety of fields, including psychology (e.g. Edelman, 1998; Shepard, 1987), information retrieval (e.g. Salton et al., 1975), and natural language processing (e.g. Deerwester et al., 1990). The set of vectors in individual information spaces can be analyzed as the geometry of those vectors, namely the set of pairwise distances between vectors. The vector geometry of information in cortical spaces is highly similar across brains (Connolly et al., 2016; Connolly et al., 2012; Connolly et al., 2011; Diedrichsen and Kriegeskorte, 2017; Guntupalli et al., 2018; Guntupalli et al., 2016; Kriegeskorte and Kievit, 2013; Nastase et al., 2017). Prior to hyperalignment, these functional pattern vectors are instantiated in the spatially organized cortical topography of a given individual. Hyperalignment leverages the inter-individual similarity of vector geometry to create a shared information space that minimizes differences between corresponding vectors across individuals while preserving vector geometry.

## Hyperalignment: Joint modeling of a common information space and idiosyncratic topographies

### A common, high-dimensional information space

The cortical feature spaces for individual brains are difficult to compare because of the inter-individual variability of topographic surface structure. The goal of hyperalignment is to create a common information space in which the vectors that encode information that is shared across brains – representations of the same stimulus or cognitive process, functional connections to other cortical fields – are aligned. Note that the goal is not simply to align the cortical patterns that carry that information in a two- or three-dimensional anatomical space (Conroy et al., 2013; Robinson et al., 2014; Sabuncu et al., 2010). The columns of data matrices in the common model space are the coordinate axes or dimensions for that information space. The rows of data matrices in the common space contain cortical information patterns that have been projected into the model space from individual data matrices. The solution to this problem requires finding individual-specific transformations, *R_i_*, that project individual data matrices, *B_i_*, in which the coordinate axes are cortical locations, into a common model coordinate space, in such a way that minimizes inter-individual differences among pattern vectors that represent the same perceptual, cognitive, or connectivity information. This is accomplished by minimizing the difference between patterns in the transformed individual data matrices, *B_i_R_i_*, and the group mean patterns, *M*. Formally,

(1) $$M=\left(1/N\right)\sum_{i=1}^{N}B_{i}R_{i}\;where\;argmin_{R}\sum_{i=1}^{N}{\left\|B_{i}R_{i}-M\right\|}_{F}$$

The dimensions in the common model space are no longer anatomical locations but, rather, are weighted sums of nearby anatomical locations in individual brains; the transformations are not a one-to-one mapping from cortical loci to common space dimensions. The distribution of these weights is specific to each brain. Each column of the transformation matrices, *R_i_*, contains the weights for this transformation for one model dimension, *d*, across all cortical loci, *v*. The transformation matrices have the same structure whether derived from response profiles or connectivity profiles. These weights model how shared information can be instantiated in idiosyncratic fine-grained cortical functional topographies.

The number of dimensions in the common space (*m* in Figure 2) can differ from the number of cortical loci in the data matrices (*k* in Figure 2) from which the space is derived. In some implementations, the number of model dimensions matches the number of cortical loci in a ‘reference brain’, allowing patterns in the model space to be illustrated in the reference brain anatomy (Guntupalli et al., 2018; Guntupalli et al., 2016; Haxby et al., 2011). In other implementations, the model space is reduced to capture shared information in a smaller number of dimensions and reduce noise or overfitting (Chen et al., 2015; Guntupalli et al., 2016; Haxby et al., 2011). Patterns in reduced dimension model spaces can be illustrated by projection into the anatomy of individual brains, using the transposes of individual transformation matrices, *R_i_*^T^.

The goal of the common information space is to align cortical pattern vectors across individuals while preserving the geometry of those vectors. The coordinate axes for that space – the model dimensions – carry information in the form of shared functional profiles (response tuning vectors or functional connectivity vectors), but they can be arbitrarily rotated, preserving vector geometry while altering the response tuning and connectivity profiles for the coordinate axes. Thus, the information associated with each common model dimension serves only to preserve the information encoded in vector geometry, shifting attention from the traditional focus on univariate response and connectivity profiles for single cortical loci to the vector geometry of distributed population responses and population connectivity that span a cortical field. The individual transformation matrices afford projecting individual data embedded in idiosyncratic cortical topographies into the common space and, conversely, projecting group data embedded in the common space into the cortical topographies of individual brains.

### Methods for deriving transformation matrices

There are various ways to derive transformations that minimize residuals around group mean functional patterns. We have concentrated on using our adaptation of the Generalized Procrustes Analysis (GPA; Gower, 1975; Guntupalli et al., 2018; Guntupalli et al., 2016; Haxby et al., 2011; Schönemann, 1966) to derive the transformation matrices. The Procrustes transformation is the orthogonal matrix that minimizes the distances between two matrices of paired vectors with a rigid rotation, which is ‘improper’ in that it allows reflections as well as rotations. GPA finds transformation matrices for a group of data matrices that minimize distances by iteratively aligning each new matrix to the mean of previously aligned matrices. A rigid rotation preserves all pairwise distances between pattern vectors within each original matrix. In the case of fMRI data matrices, therefore, this transformation preserves the vector geometry of each data set (representational geometry or connectivity geometry [Connolly et al., 2016; Connolly et al., 2012; Connolly et al., 2011; Guntupalli et al., 2018; Guntupalli et al., 2016; Kriegeskorte et al., 2008a; Kriegeskorte and Kievit, 2013]). We and others have shown that local vector geometry is highly correlated across brains (Connolly et al., 2016; Connolly et al., 2012; Connolly et al., 2011; Guntupalli et al., 2018; Guntupalli et al., 2016; Kriegeskorte and Kievit, 2013; Nastase et al., 2017), indicating that it is a key feature of shared information structure (Diedrichsen and Kriegeskorte, 2017; Ejaz et al., 2015). Hyperalignment algorithms that mostly preserve vector geometry tend to generalize better to novel data than algorithms that do not (Xu et al., 2012).

### Searchlight-based whole-cortex hyperalignment

**Figure 3.**
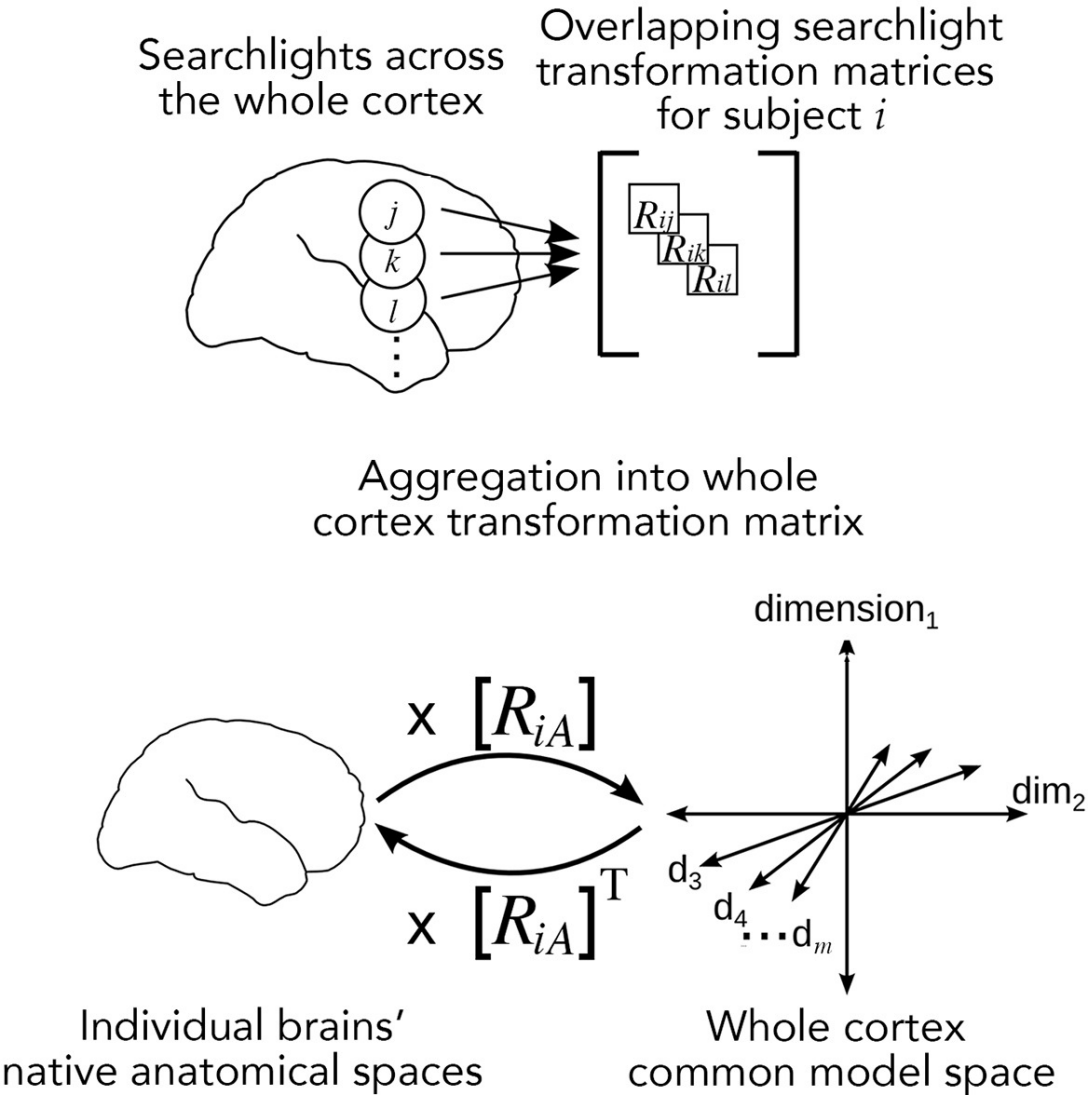
Searchlight hyperalignment for building a common model and transformation matrices for the whole cortex. Overlapping transformation matrices (*R_ij_*, *R_ik_*, …) are calculated for overlapping searchlights in each subject and aggregated into a single whole-cortex transformation matrix, *R_iA_*. The transpose of the transformation matrix, *R_iA_*^T^, allows pattern vectors from the common model space to be projected into an individual brain’s topographies. The aggregation step results in a whole-cortex transformation that is mostly orthogonal locally (Guntupalli et al., 2016) but globally nonorthogonal.

Hyperalignment remixes data across spatial loci; however, for whole-cortex hyperalignment, we want to avoid remixing information from distant voxels. We have developed whole-cortex hyperalignment algorithms using GPA that mostly preserve local vector geometry but not the orthogonality of the whole-cortex transformation matrices (Figure 3; Guntupalli et al., 2018; Guntupalli et al., 2016). This was necessary to impose a locality constraint whereby information from any cortical location was resampled only into nearby locations in the ‘reference’ cortical anatomy. Local vector geometry also can be preserved by restricting hyperalignment to regions of interest (ROIs; Chen et al., 2015; Haxby et al., 2011) that tessellate the cortex (e.g., Glasser et al., 2016; Gordon et al., 2016; Shen et al., 2013; Shen et al., 2010), but this may impose an excessively restrictive prior constraint, as the topographies associated with more unconstrained model dimensions usually do not conform to areal boundaries (Haxby et al., 2011). Whole-cortex searchlight hyperalignment, by contrast, makes local models in overlapping searchlights (Kriegeskorte et al., 2006; Oosterhof et al., 2011) that cover the cortex, then aggregates the local transformation matrices into a whole-cortex transformation matrix, thereby mostly preserving local vector geometry (Guntupalli et al., 2016). Data are aligned anatomically to a standard cortical surface (e.g., FreeSurfer's fsaverage; Fischl et al., 1999) prior to whole-cortex searchlight hyperalignment. The cortical fields for local basis functions, defined by the voxels with nonzero weights in columns of the whole-cortex transformation matrices, are overlapping and constrained only by searchlight size, not by areal boundaries. Searchlights of increasing radius will accommodate larger divergence in individual topographies at the expense of spatial specificity; the ‘appropriate’ searchlight size may vary across brain areas and experiments, and this has not yet been systematically examined. Boundaries of cortical areas can be constructed from weighted sums of these local basis functions but appear to play a minor role in our model of cortical topographies.

### A cortical field in the whole-cortex common model

A cortical field of contiguous cortical vertices, such as a searchlight or ROI, in the common information space produced by whole-cortex hyperalignment is only defined insofar as it is instantiated in the idiosyncratic cortical topography of an individual brain. Consequently, a study of local function with hyperaligned data requires projecting individual data into one subject’s cortical anatomy, referred to as the reference subject. Other subjects’ data in a cortical field in the reference subject’s anatomical space are idiosyncratic mixtures of vertices from the same cortical field, anatomically-defined, as well as nearby vertices. Thus, data that is more similar to other subjects’ data in a given cortical field is retained and imported from nearby cortex, whereas data that is more similar to that of others’ data in nearby cortical fields is resampled out and into those other locations. The result of this resampling of information into and out of cortical fields is that the local individual information spaces in cortical fields are more consistent across brains, as evidenced, for example, by higher intersubject correlations of local vector geometry. Using a different subject’s anatomy as the reference results in similar alignment of local information spaces with a different distribution of how information spaces vary across cortical fields.

### Reducing the dimensionality of the common model space

The functional profiles of cortical loci are often redundant, and the information encoded in the vector geometry for a given cortical field may be lower-dimensional than the number of constituent cortical loci. We have shown that reducing the dimensionality of the common model space using principal component analysis (PCA; Guntupalli et al., 2016; Haxby et al., 2011) can preserve or even improve performance, as indexed with between-subject multivariate pattern classification (bsMVPC; Guntupalli et al., 2016; Haxby et al., 2011). Later work suggests that bsMVPC accuracies can be substantially higher with lower dimensional models (Chen et al., 2015). Dimensionality reduction necessarily warps vector geometry but can effectively denoise data in common space by discarding lower-order eigenvectors.

### Other algorithms for hyperalignment

We and others also have developed algorithms other than GPA for deriving transformation matrices in individual brains, such as regularized canonical correlation analysis (rCCA; Bilenko and Gallant, 2016; Xu et al., 2012), joint singular vector decomposition (SVD; Chen et al., 2014), and probabilistic estimation (Chen et al., 2015). With regularized CCA we find that a small relaxation of the orthogonality constraint enhances performance, as indexed with between-subject multivariate pattern classification (bsMVPC). Chen et al., 2015 probabilistic approach, the Shared Response Model (SRM), pre-specifies a lower dimensionality for the common model, resulting, as noted above, in a warping of individual representational geometry that appears to filter out noise and reduce overfitting.

In our discussion of the effectiveness of hyperalignment, we will concentrate on results from analyses using the GPA-based algorithms. Optimization of the methods for deriving transformation matrices, however, is an ongoing subject of investigation.

### Connectivity hyperalignment

The hyperalignment algorithm can also be used to construct a common model based on functional connectivity patterns. Previous work has shown that the human connectome has fine-grained topographies that are not captured by mean connections between areas (Arcaro et al., 2015; Braga et al., 2019; Haak et al., 2018; Heinzle et al., 2011; Jbabdi et al., 2013) and connectivity hyperalignment has shown that this fine-scale structure in the connectome is prevalent across all of cortex and of higher dimensionality than can be captured with known topographies, such as retinotopy and orientation selectivity (Guntupalli et al., 2018).

Connectivity hyperalignment requires a matrix of connectivity pattern vectors, in which each column is a cortical location in the cortical region to be hyperaligned and each row is a pattern of connectivities with a target location elsewhere in the brain (Guntupalli et al., 2018). Connectivity targets can be the responses for a single location (e.g., a cortical vertex), the mean response time-series for a cortical area or searchlight, or one or more component of the time-series in a cortical area after decomposition (e.g., using PCA). Once individual connectivity matrices are calculated, connectivity hyperalignment proceeds in the same way as response hyperalignment. Iteratively repeating connectivity hyperalignment allows connectivity target time-series to be rearranged and recalculated. Whereas response hyperalignment depends on response pattern vectors in experimental data that are locked to stimuli, connectivity hyperalignment depends on connectivity vectors for a defined set of connectivity targets and does not require a common stimulus paradigm, affording hyperalignment of resting state data (Guntupalli et al., 2018) or across different experiments (Nastase et al., 2020).

For a cortical field, the pattern of functional connectivity to a target can be thought of as a population connectivity vector, analogous to thinking of the pattern of activity evoked by a stimulus as a population response vector. Thus, while response hyperalignment aligns population responses across brains, preserving vector geometry of representation, connectivity hyperalignment aligns population connectivity vectors across brains, preserving their vector geometry. These two algorithms, however, mostly converge, both producing a high-dimensional common model space with individual transformation matrices that project individual anatomical spaces into a model space. Transformation matrices derived with connectivity hyperalignment can be applied to response data, effectively aligning response pattern vectors (Guntupalli et al., 2018; Nastase et al., 2020). Validation tests show that response hyperalignment aligns connectivity information and connectivity hyperalignment aligns population response information.

### Rich, naturalistic stimuli

Estimating the parameters to transform high-dimensional spaces from individual brains into a common high-dimensional space requires a rich set of data that samples a wide variety of cortical patterns in order to generalize to novel stimuli or tasks. For response hyperalignment, a rich variety of stimuli or conditions are necessary to sample the response vector space. For connectivity hyperalignment, the sampling of connectivity vector space is defined by the selection of connectivity targets, but the richness and reliability of connectivity estimates depends on the variety of brain states over which connectivity is estimated.

We have shown that a common model space based on transformation matrices derived from responses to naturalistic audiovisual movies has general validity across a variety of experiments (Guntupalli et al., 2016; Haxby et al., 2011; Nastase et al., 2017). By contrast, transformation matrices estimated from controlled experiments with a limited set of stimuli or cognitive states may suffice for that experiment, but do not generalize well to other contexts or paradigms (Haxby et al., 2011).

The general validity that responses to naturalistic movies afford may stem from several important attributes of movies (Hasson et al., 2010; Hasson et al., 2004; Haxby et al., 2020). Such stimuli evoke a rich variety of brain states involving multiple coordinated systems for visual and auditory perception, perception of dynamic action, narrative, and speech, memory, attention, semantic knowledge, and social cognition. Movies are engineered to be engaging and guide attention. Movies also build a rich set of expectations for upcoming stimuli and events affording strong predictions (and prediction errors). Controlled experiments, on the other hand, typically randomize trial order, deliberately decontextualizing each event and providing no basis for accurate prediction. Consequently, randomization of trial order nullifies the utility of neural information processing related to the formation and application of predictive coding, making prediction-related activity weak and inconsistent across brains. In a comparison of between-subject multivariate pattern classification (bsMVPC) of responses to dynamic stimuli in movies to bsMVPC of responses to static stimuli in controlled experiments, with the structure of these analyses carefully matched to allow meaningful comparison, bsMVPC accuracies in ventral temporal extrastriate visual cortex for naturalistic, dynamic stimuli were twice that for unpredictable static stimuli (Supplemental Figure 4A in Haxby et al., 2011), indicating a substantial advantage of these stimuli for evoking distinctive brain states.

A common model space also can be built by estimating transformation matrices from fMRI data collected in the resting state (rs-fMRI) using connectivity hyperalignment (Guntupalli et al., 2016). Such a model greatly increases intersubject correlations of functional connectivity indices and captures the fine-scale spatial structure of variation in connectivity that is seen in individual data. The variety of brain states that are sampled in resting state paradigms is difficult to determine. The general validity of a common model based on rs-fMRI data for aligning brain states associated with a wide variety of stimuli and cognitive processes has not yet been established.

## A new conceptual basis for cortical functional topographies

Previous models of cortical functional architecture have focused on the division of cortex into contiguous functional areas (Brodman, 1909; Glasser et al., 2016; Gordon et al., 2016; Shen et al., 2013; Shen et al., 2010; Yeo et al., 2011; Zilles and Amunts, 2010). These parcellations, however, may vary substantially across functional brain state (Salehi et al., 2020) and individuals (Braga and Buckner, 2017; Gordon et al., 2017; Kong et al., 2019). The conceptual framework for hyperalignment proposes a radically different basis for cortical topographies that is based on overlapping topographic basis functions, offering a model that accounts for individual variation in both fine-scale topographies and coarse-scale areal structure.

### Individual cortical topography as a composite of common model pattern bases

Hyperalignment transformation matrices provide the basis for a new model for the organization of cortical topographies. Each column in an individual transformation matrix (*R_i_*) (or row in the transpose of that matrix, *R_i_^T^*), is a pattern vector of weights for cortical loci in an individual subject’s brain. These pattern vectors for model dimensions serve as topographic basis functions. A cortical response or connectivity pattern in an individual brain ($t_{p(B_{i})}$) is modeled as a weighted sum of these basis functions, using the dimension values for the pattern vector in the model group data matrix ($t_{p(M)}$) as weights:

(2) $${\hat{t}}_{P}(B_{i})=t_{p(M)}R_{i}^{T}$$

**Figure 4.**
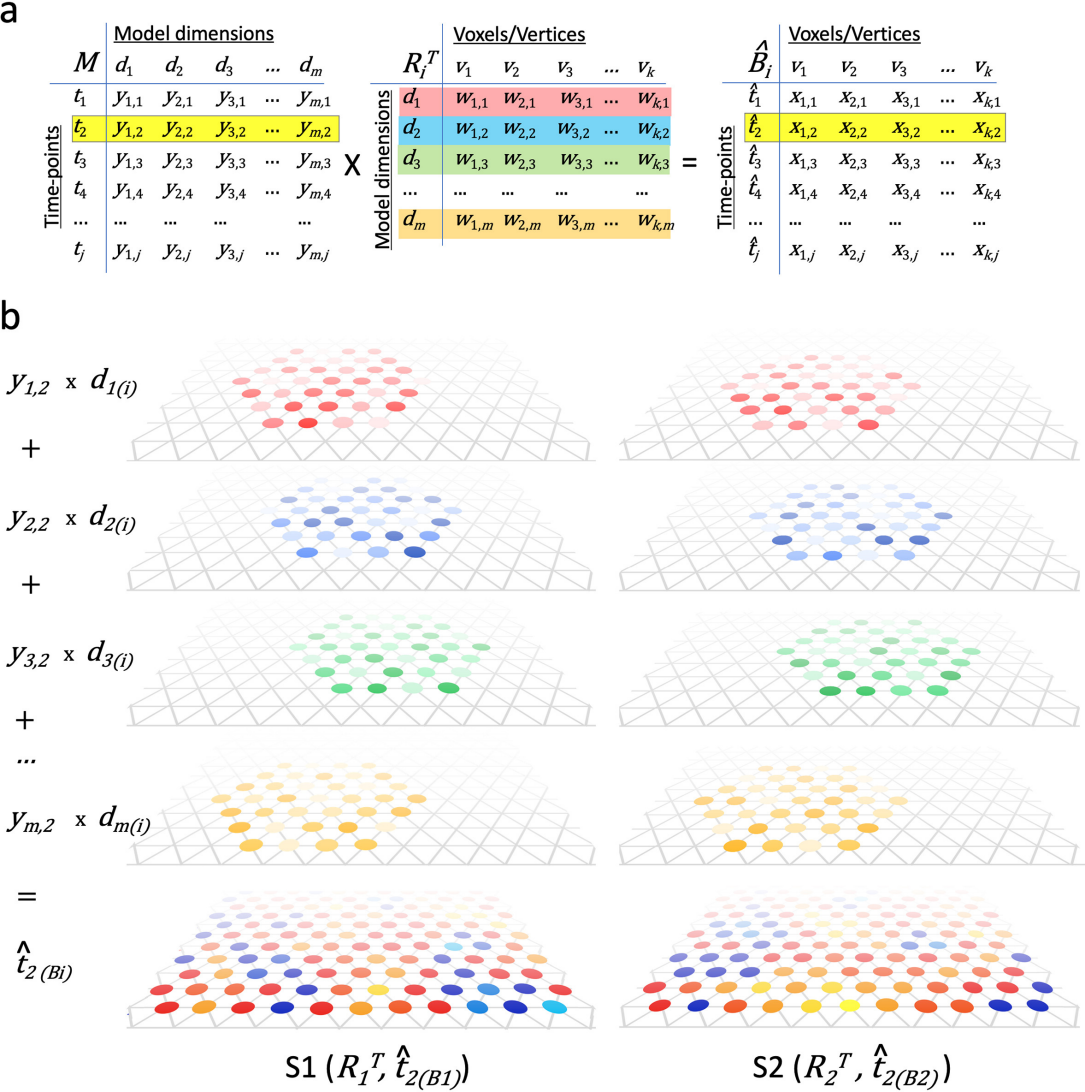
Modeling individual cortical patterns using model topographic basis functions. (**a**) Matrix multiplication for modeling cortical patterns in individual brains (*t*_2(Bi)_) by multiplying a pattern vector (t_2_) in the model space (M) and the transpose of individual-specific transformation matrices (*R_i_^T^*). (**b**) Illustration of topographic basis functions in two subjects, S1 and S2, for model dimensions (*d_1_, d_2_, d_3_, …, d_m_*), which are comprised of weights (*w*’s in *R_i_^T^*) across vertices in overlapping but non-coextensive patches of cortex. This image is illustrative and not based on experimental data. Two hypothetical subjects’ basis functions are illustrated to emphasize that these functions are individual-specific and support the same shared information from *M* in variable topographic patterns in individual brains. Transformation matrix weights (w) are illustrated as colored circles of varying intensity at vertices of a stylized triangular cortical mesh. The pattern of weights for each basis function varies across brains. These topographic basis functions are combined as a weighted sum, using the weights from a pattern vector (e.g., t_2_) in the model data matrix (*y_1,2_, y_2,2_, y_3,2_, …, y_m,2_* in *M*) to model or predict a topographic pattern (*t̂_2(Bi)_*) in an individual subject’s cortex. The same weights in the model pattern vector predict different patterns of response in each individual brain. The predicted topographic patterns (*t̂_2(Bi)_*) are illustrated as values at each vertex of a triangular cortical mesh using a typical color scale with negative values in shades of blue and positive values in shades of red, orange, and yellow.

Thus, the topographies of cortical patterns are modeled as overlaid, weighted topographic basis functions that are individual-specific and formalized as the columns in transformation matrices (Figure 4). This framework captures how shared information from the common model space can be instantiated in individually-variable cortical topographies using a computationally-specified linear model with basis functions that can be estimated using hyperalignment.

### Modeling response patterns and known topographies

This conceptual framework affords estimating the location of functional areas and the conformation of functional topographies in a given subject based on data from other subjects. The model constructed by hyperalignment allows us to transfer fine-scale cortical functional topographies across individuals via the common information space. Patterns that specify an area or topographic pattern, such as a face-responsive area or retinopy, in other subjects’ cortex (*t_p(B)_*) can be transformed into a group mean pattern in the common model information space (*t_p(M)_*) using their transformation matrices:

(3) $$t_{p(M)}=(1/N)\sum_{i=1}^{N}t_{p(B_{i})}R_{i}$$

The mean topographic pattern in the model space (*t_p(M)_*) can then be projected into a new individual’s cortical space using that individual’s transformation matrix and Equation 2, providing an estimate of that pattern (*t̂_p(Bi)_*) in that individual’s brain.

## Evaluating the efficacy of hyperalignment

Assessing the performance of hyperalignment for modeling shared information and idiosyncratic topographies requires testing on data that play no role in calculating the parameters in the transformation matrices (Kriegeskorte et al., 2009). Generally appropriate cross-validation is achieved by dividing the data for deriving hyperalignment – usually movie-viewing or resting state data – into training and test folds. If test data are from an unrelated experiment, the full movie-viewing dataset can be used for calculating hyperalignment parameters.

### Three ways to assess shared information: Patterns, profiles, and geometry

Hyperalignment of individual data spaces defined by cortical loci into a common model space is designed to maximize intersubject similarity of information content as represented by data matrices (Equation 1). Similarity can be indexed in terms of intersubject similarity of patterns for time-points, stimuli, or connectivity targets (rows in the data matrices), intersubject similarity of functional profiles (columns in the data matrices), or intersubject similarity of vector geometry (covariance or dissimilarities between rows in the data matrices; see Nastase et al., 2019, for a recent review). Numerous tests with different data sets have shown marked and robust improvement of intersubject similarity on all of these indices (e.g., Chen et al., 2015; Guntupalli et al., 2018; Guntupalli et al., 2016; Haxby et al., 2011; Nastase et al., 2017; Van Uden et al., 2018), confirming that the common model space captures shared information across individuals. Here, we present a sampling of these results to convey the size of these effects and the general validity of models built with hyperalignment.

### Between subject multivariate pattern classification (bsMVPC)

**Figure 5.**
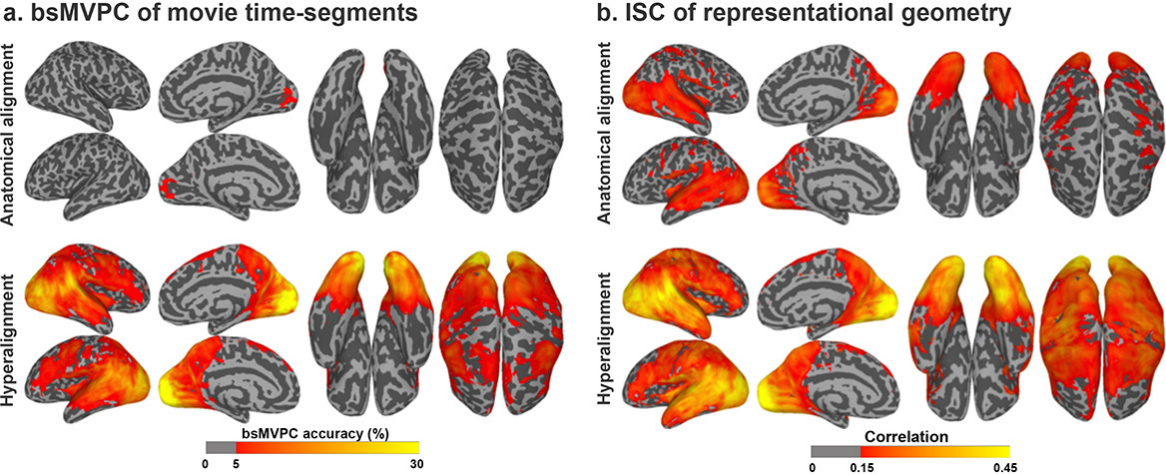
Enhancement of between-subject multivariate pattern classification (bsMVPC) and intersubject correlation (ISC) of representational geometry with hyperalignment. (**a**) Accuracy of bsMVPC of movie time segments from anatomically-aligned and hyperaligned data (reproduced from Figure 2, Guntupalli et al., 2016). Chance performance is less than 0.1%. (**b**) ISC of representational geometry for movie time points (reproduced from Figure 3, Guntupalli et al., 2016). Representational geometry for a movie is calculated as the matrix of pairwise similarities between responses to different time-points (>1300 time-points; >800,000 pairs).

The goal of hyperalignment is to find transformations that cluster different subjects’ pattern vectors more tightly, thus making each individual subject’s vectors more classifiable based on other subjects’ vectors. In one test, which is particularly demanding and dependent on fine-scale topographies, response patterns for short time-segments of a movie are classified relative to other time-segments of the same movie (Figure 5a; Chen et al., 2015; Guntupalli et al., 2018; Guntupalli et al., 2016; Haxby et al., 2011; Xu et al., 2012). The magnitude of the beneficial effect of hyperalignment on bsMVPC is largest for small and moderately-sized ROIs which contain fine-scale topographies with minimal coarse-scale variation, where bsMVPC accuracies for anatomically-aligned data are very low. In small cortical regions, where accuracies for unsmoothed, anatomically aligned data are very low, hyperalignment provides more than a full order of magnitude improvement in bsMVPC (Guntupalli et al., 2018; Guntupalli et al., 2016). Accuracies are higher in larger, more heterogeneous regions for both anatomically and hyperaligned data, but hyperalignment still yields markedly higher bsMVPC accuracies (Guntupalli et al., 2016; Haxby et al., 2011), highlighting the role of information encoded in fine-scale structure that cannot be captured by anatomical alignment, and the importance of aligning this fine-scale information in building MVP models that generalize across subjects.

### Intersubject correlations (ISCs) of response and connectivity profiles

**Figure 6.**
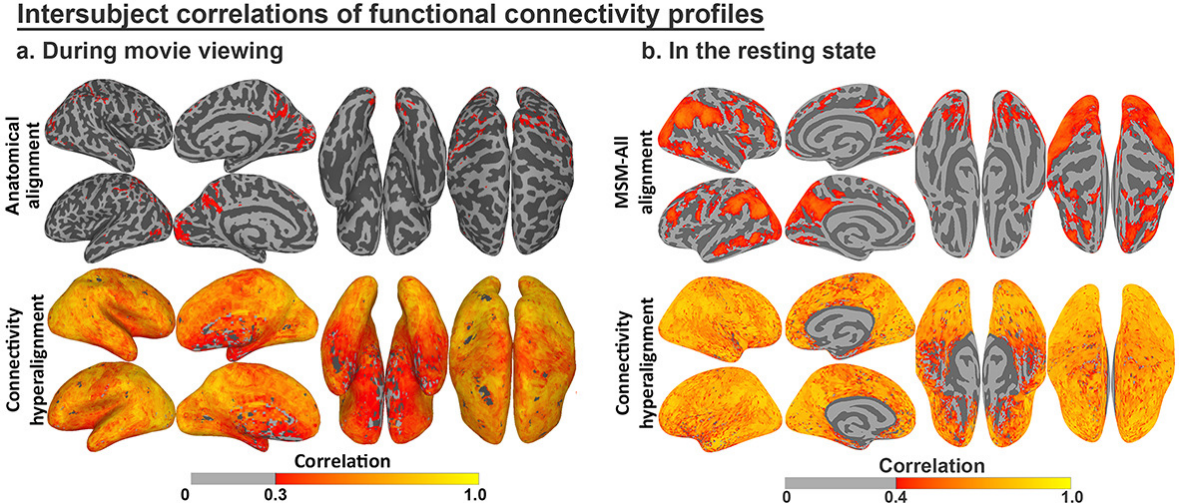
Enhancement of intersubject correlation (ISC) of functional connectivity profiles after connectivity hyperalignment. (**a**) ISC of connectivity profiles measured during movie viewing (reproduced from Figure 3, Guntupalli et al., 2018). (**b**) ISC of connectivity profiles measured in the resting state (reproduced from Figure 4, Guntupalli et al., 2018).

The functional profiles for anatomically-aligned cortical loci (vertices) and hyperaligned model dimensions also can be evaluated for between-subject consistency (Guntupalli et al., 2018; Guntupalli et al., 2016; Hasson et al., 2004). Modeling response and connectivity profiles using hyperalignment markedly increases ISCs of both (Guntupalli et al., 2018; Guntupalli et al., 2016). For example, response hyperalignment nearly doubled ISCs of time-series responses to a naturalistic movie (Guntupalli et al., 2016), and ISC increases were even greater for functional connectivity profiles after connectivity hyperalignment, for both functional connectivities measured during movie viewing and during the resting state (Figure 6; see Guntupalli et al., 2018, for details).

### Vector geometry

Vector geometry provides an integrative measure of information in an information space in the form of all pairwise similarities, or distances, between pattern vectors (Diedrichsen and Kriegeskorte, 2017; Haxby et al., 2014; Kriegeskorte et al., 2008a; Kriegeskorte and Kievit, 2013). For response data matrices, vector geometry reflects representational geometry – the similarities among response patterns to different stimuli or conditions. For connectivity data matrices, vector geometry reflects connectivity geometry – the similarities among connectivity patterns for a set of connectivity targets. In addition to its incorporation of information from a full data matrix, between-subject similarity of vector geometry explicitly captures information that is not reflected in between-subject similarity of cortical patterns or between-subject similarity of response or connectivity profiles (Kriegeskorte et al., 2008a; Kriegeskorte et al., 2008b). Comparison of vector geometries for different cortical fields also provides a window on how information spaces are transformed along processing pathways, enhancing relevant distinctions and diminishing confounding information (Connolly et al., 2016; Guntupalli et al., 2017; Kriegeskorte and Kievit, 2013; Nastase et al., 2017).

Searchlight-based whole-cortex hyperalignment greatly increases ISCs of local representational geometry (Figure 5b; Guntupalli et al., 2018; Guntupalli et al., 2016; Nastase et al., 2017; Van Uden et al., 2018). For naturalistic movies, hyperalignment increased mean ISC of representational geometry across searchlights by 46% to 75% (Guntupalli et al., 2016; Van Uden et al., 2018). Simpler representational geometries in an experiment with 20 stimulus conditions showed similar increases in ISCs after data were hyperaligned based on responses to an unrelated movie (Nastase et al., 2017).

Increased ISC of vector geometry is counterintuitive and, therefore, instructive about cortical functional architecture. Procrustes-based hyperalignment preserves local vector geometry with rigid rotation of the high-dimensional pattern vectors. Indeed, ROI hyperalignment does not change the vector geometry for each individual and, therefore, has no effect on ISC of vector geometry. Searchlight hyperalignment, by contrast, resamples information into and out of local cortical fields, thereby increasing the similarity of local information content across subjects. The size of the increase suggests this is a large effect and that the dissimilarity of vector geometry for overlapping cortical fields is substantial. RSA of anatomically-aligned data, therefore, does not capture most of the shared fine-scale variation in local vector geometry of cortical fields along processing pathways that may be a key to understanding how information is processed and transformed.

### General validity of common model information spaces

The shared functional profiles and preserved vector geometry in common model information spaces have a general validity that extends beyond the specific stimuli used to derive them. All of the validation tests described in the previous sections derived the common model spaces and transformation matrices using data that were independent of the data used to test bsMVPC or ISC of response profiles, connectivity profiles, and vector geometry (Kriegeskorte et al., 2009). For movie data, this involved using data from different parts of the movie for model derivation and validation. For the resting state, model derivation and validation were performed on different resting state sessions. The generalizability of the validity of common model spaces based on naturalistic movie viewing and the resting state also extends to experiments with very different structure. For example, bsMVPC of responses to still images of objects, faces, and animals after data were projected into a common space based on responses to a movie was equivalent to or better than within-subject MVPC (Guntupalli et al., 2016; Haxby et al., 2011). Higher bsMVPC than within-subject MVPC of hyperaligned data demonstrated the added power of using large multi-subject data sets for training a pattern classifier, which hyperalignment makes possible. Similarly, ISC of representational geometry for responses to brief video clips of different categories of animals performing different actions were greatly increased after data were projected into a common space based on responses to a continuous movie (Nastase et al., 2017). Moreover, connectivity hyperalignment now makes it possible to derive transformation matrices to project new subjects’ cortical space into a model space using connectivity data from experimental paradigms (e.g., a movie or the resting state) that differ from the experimental paradigm used to derive that common model space (Guntupalli et al., 2018; Nastase et al., 2020). In a novel application (Taschereau-Dumouchel et al., 2018), hyperalignment made it possible to create a response pattern template based on other subjects’ hyperaligned data for use in new subjects who were never exposed to the stimulus as a target brain state for neurofeedback.

### Generalization across response and connectivity hyperalignment models

Response and connectivity hyperalignment both produce transformation matrices that project data from cortical coordinate spaces into a common model information space. The two algorithms align the vector geometries for related, but distinct types of information – patterns of responses and patterns of connectivity. Both algorithms produce transformations that can be used to align either type of data (Guntupalli et al., 2018), but the common model spaces, while closely related, are not equivalent (Table 1). Response hyperalignment of movie data increased mean intersubject correlation of connectivity profiles from 0.15 to 0.58, relative to anatomical alignment, but ISC of connectivity profiles are even higher after connectivity hyperalignment (mean ISC = 0.67). Connectivity hyperalignment of the same data increased mean intersubject correlation of representational geometry from 0.21 to 0.31 and bsMVPC of movie time segments from 1.0% to 10.4%, relative to anatomical alignment. Response hyperalignment, however, produced even higher ISC of representational geometry (mean ISC = 0.32) and bsMVPC accuracies (mean = 13.7%). Thus, aligning shared representational vector geometry also aligns connectivity vector geometry, but this response-based hyperalignment of connectivity, and the underlying group mean connectivity basis functions associated with model dimensions, is suboptimal. Similarly, aligning shared connectivity vector geometry also aligns representational vector geometry, but connectivity-based representational alignment, and the underlying group mean response tuning basis functions associated with model dimensions, is suboptimal. At this point it is unclear if this discrepancy between models of shared population codes and shared connectivity is fundamental or will converge to a common solution with better data and algorithms.

**Table 1.** Summary of improvement in indices of performance after response hyperalignment (RHA) and connectivity hyperalignment (CHA), relative to anatomical alignment (AA). ISC: Intersubject correlation; bsMVPC: between subject multivariate pattern classification; >: small advantage; >>>: large advantage.

Performance measure	Relative performance after different alignments
ISC of searchlight representational geometry	RHA > CHA >>> AA
bsMVPC of movie time segments	RHA > CHA >>> AA
ISC of vertex connectivity profiles	CHA > RHA >>> AA

### Estimating individual cortical topographies

**Figure 7.**
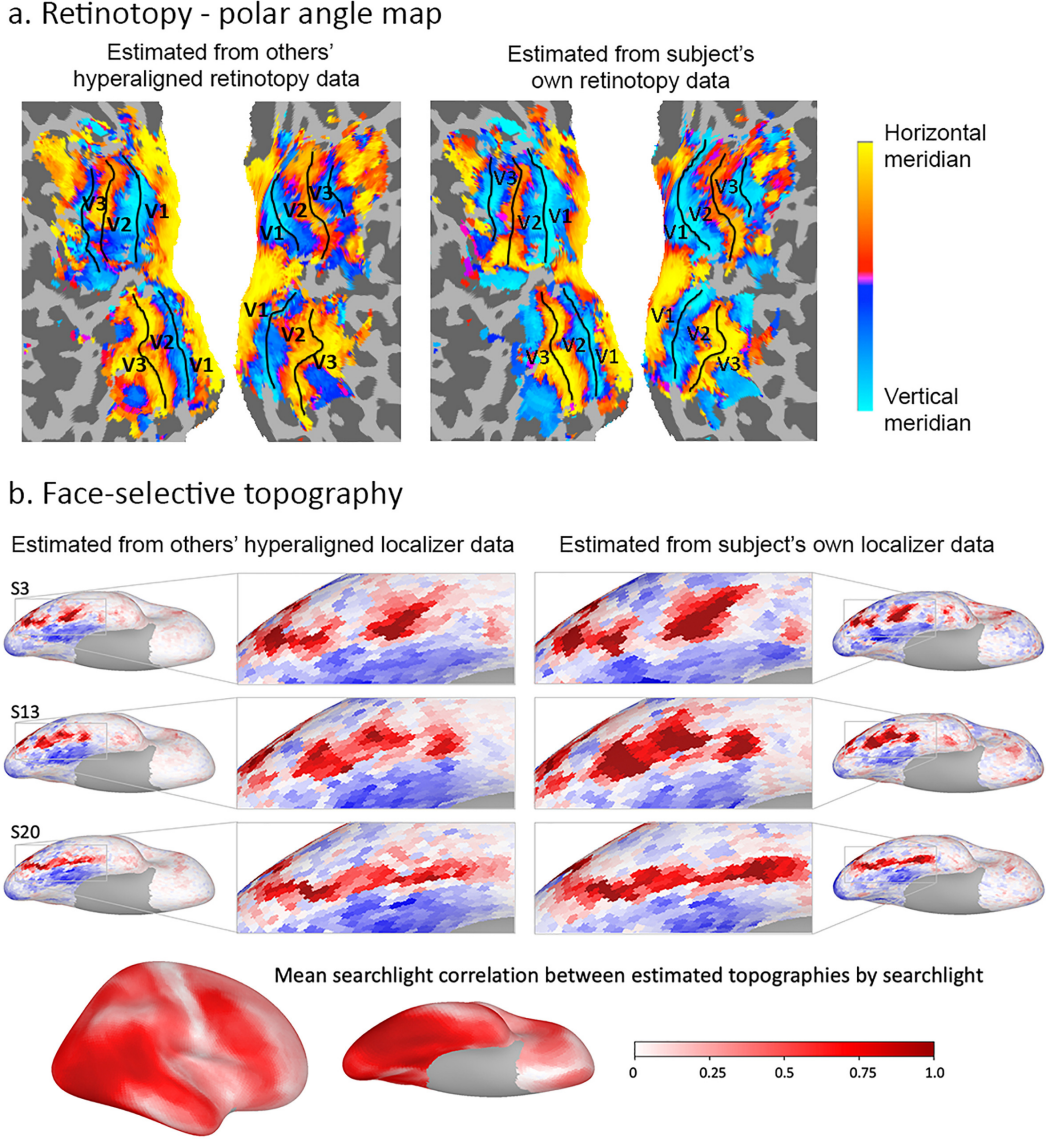
Estimation of functional topographies in individual brains based on data from other brains that are projected into an individual’s cortical space using transformation matrices calculated with hyperalignment based on data gathered while subjects watched a movie. (**a**) Retinotopic map of polar angle based on other subjects’ retinotopy localizer fMRI data projected into a subject’s visual cortex (left) and based on that subject’s own retinotopy localizer fMRI data (adapted from Figure 7, Guntupalli et al., 2016). (**b**) Face-selective topography based on other subjects’ face-selectivity localizer fMRI data projected into three subjects’ cortical anatomy using hyperalignment and based on their own localizer data (from Jiahui et al., 2019). Ventral views of the right hemisphere with enlarged images of occipital and posterior temporal cortices. Local correlations in searchlights between estimates based on others subjects’ data and estimates based on own data exceeded 0.80 in posterior ventral temporal, lateral occipital, and posterior superior temporal cortices and strong correlations extend into anterior ventral temporal, anterior superior temporal sulcal and lateral prefrontal cortices (lower image). Subject numbers match those in Supplementary Figure 2 in Jiahui et al., 2019.

**Figure 8.**
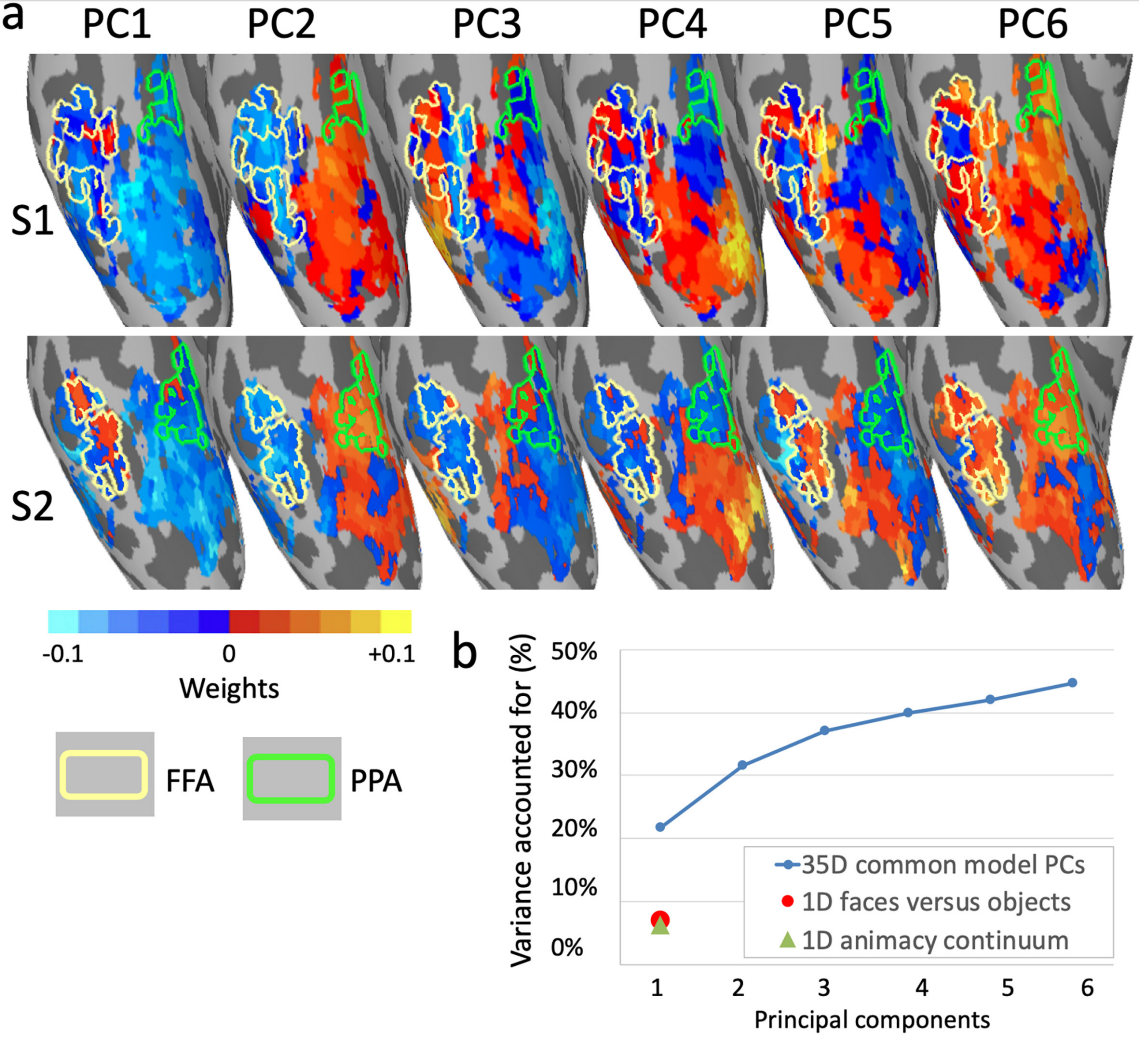
Topographic basis functions and comparison to one-dimensional models of functional topography. (**a**) Topographic basis functions for two subjects, S1 and S2, for the top six principal components (PCs) in a common model of ventral temporal cortex and the linear discriminant, a weighted sum of PCs, for the contrast faces versus objects (from Haxby et al., 2011; only the right hemisphere cortex is shown here). The locations of the fusiform face area (FFA) and parahippocampal place area (PPA) are indicated with yellow and green outlines, respectively. (**b**) Cumulative variance accounted for by the top six PCs in a 35-dimensional common model compared to the variance accounted for by one-dimensional linear discriminants for face selectivity (responses to faces versus houses) and for the animacy continuum (responses to primates versus insects).

Individual-specific functional topographies, the locations and borders of category-selective regions in ventral temporal cortex, and the retinotopic topography of early visual cortex can be estimated with high fidelity based on other subjects’ topographies with hyperalignment (Figures 7 and 8; Guntupalli et al., 2016; Haxby et al., 2011; Jiahui et al., 2019).

### Overlaid local topographic basis functions and areal boundaries

The model coordinate axes can be rotated arbitrarily to model individual topographies while preserving vector geometry. Modeling functional areal boundaries is essentially a rotation of coordinate axes in common space to align a contrast or continuum that defines a functional area (e.g., faces versus objects linear discriminant) or topography (e.g., retinotopic polar angle) with a single axis, then plotting the weights of the topographic bases for that axis. Aligning common model coordinate axes to well-understood functional dimensions can make the model more interpretable, but how much of the information that is encoded in fine-scale topographies is captured by such dimensions?

Principal components analysis affords quantification of how much information in a dataset can be accounted for by single dimensions. The first eigenvector is defined as the axis along which variance is maximal, the second eigenvector is the orthogonal axis along which the residual variance is maximal, and so forth. Thus, PCA rotates the space to account for variance most efficiently, rather than rotating the space to fit axes to a priori functional distinctions. Applying PCA to the model space for ventral temporal cortex, developed based on patterns of activity evoked by a naturalistic movie, revealed that approximately 35 principal components (PCs) were sufficient to account for the information content of one hour of the movie, as indexed by bsMVPC (Haxby et al., 2011). This PCA allows evaluation of the roles played by dimensions defined a priori by functional distinctions relative to the information that is captured by the full model in which both coarse-grained and fine-grained topographies can be modeled as mixtures of topographic basis functions. Comparison of the topographies for the top PCs with the topography for face versus object category selective areas (Figure 8a), shows that this functional distinction does not emerge as one of the top dimensions but only as a mixture of PCs (Figure 8a rightmost images), raising the question of how large a role it plays in the functional topography of ventral temporal cortex under naturalistic conditions.

We defined an a priori dimension with the contrast between responses to faces and objects (Haxby et al., 2011), calculated as a linear discriminant in the model space (Figure 8a). Response variance for a dynamic, naturalistic movie along that dimension comprised 7% of the total variance and 12% of the variance accounted for by the 35-dimensional model (58% of the total variance in movie response data was accounted for by all 35 dimensions, cross-validated on independent data; Figure 8b). By contrast, the first PC accounted for over three times more variance (22%, cross-validated), and the first six PCs together accounted for over six times more variance. In a second analysis, the contrast between responses to still images of primates and insects (Connolly et al., 2012; Haxby et al., 2011) was calculated as a linear discriminant in model space. This dimension captures the animacy continuum and is mostly collinear with the faces versus objects dimension. The mean correlation of movie time-series for these two dimensions is 0.83, and the mean correlation of their topographies in ventral temporal cortex is 0.77. Although this animacy continuum dimension accounts for over 50% of variance in the responses to still images of animals and objects (Sha et al., 2015), it accounts for only 6% of variance in responses to the movie. Thus, some of the most intensively studied dimensions in the coarse-scale functional topography of ventral temporal cortex (Connolly et al., 2012; Epstein and Kanwisher, 1998; Grill-Spector and Weiner, 2014; Kanwisher et al., 1997; Khaligh-Razavi and Kriegeskorte, 2014; Kriegeskorte et al., 2008b; Sha et al., 2015; Thorat et al., 2019) are faithfully represented in the common model space (see Figure 7b) but play a relatively minor role in the more comprehensive, high-dimensional model of response tuning to dynamic, naturalistic stimuli and coarse- and fine-scale functional topographies.

### Spatial granularity of the common model

The common model accounts for individual functional topographies as mixtures of individual-specific topographic basis functions. The results showing bsMVPC accuracies equivalent to or better than within-subject MVPC indicate that these mixtures preserve the fine-grained patterns that carry information. Analysis of the spatial point-spread function (PSF) for response and connectivity profiles provides a more direct measure of the preservation of the granularity of cortical topographies (Guntupalli et al., 2018; Guntupalli et al., 2016).

**Figure 9.**
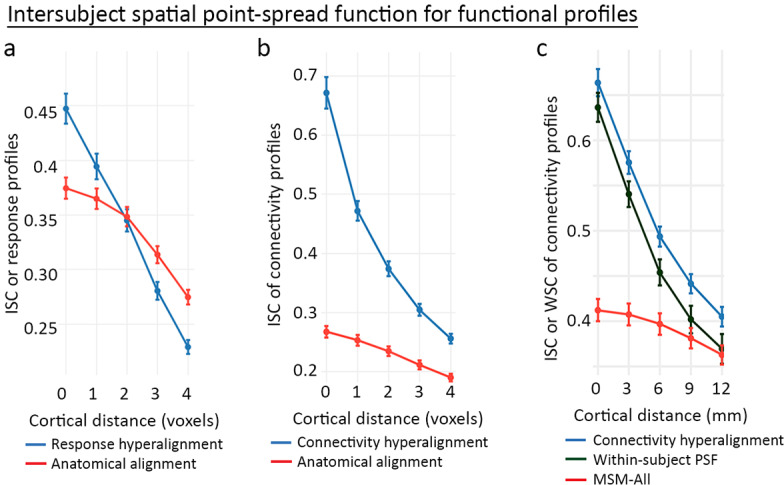
Spatial point-spread functions (PSFs) for intersubject correlations of time-series response profiles and connectivity profiles. A greater negative slope indicates more spatially fine-grained functional selectivity. (**a**) Spatial PSFs for movie data response profiles (reproduced from Figure 5, Guntupalli et al., 2016). (**b**) Spatial PSFs for movie data connectivity profiles and c. resting state fMRI connectivity profiles with comparison to within-subject PSFs based on within-subject correlations between independent resting state sessions. Error bars are standard errors (SE) across participants, in which the value for each participant is the mean across searchlight ROIs (20 ROIs in a and 24 ROIs in **b** and **c**). MSM-All alignment is multimodal surface matching (Robinson et al., 2014). (Reproduced from Figure 5, Guntupalli et al., 2018).

Spatial PSF analysis measures the intersubject correlations of response and connectivity profiles for a given cortical vertex and its neighboring vertices that differ in anatomical location by varying distances. For anatomically aligned data, intersubject correlations of profiles for neighboring loci are only slightly lower than correlations of profiles for loci at the same location. For data projected from the common model into one subject’s anatomy, the function is dramatically steeper with large differences in intersubject correlation of profiles from the same location and correlations of profiles from contiguous locations (Figure 9), indicating that the common model preserves the distinctiveness of functional profiles with a spatial granularity of a single voxel or vertex (~3 mm). This level of spatial granularity was found for all cortical fields tested in occipital, temporal, parietal, and prefrontal cortices. A within-subject PSF for connectivity profiles calculated using resting-state fMRI data from independent sessions in the same subject was equivalent to the between-subject PSF. This fine-grained structure in the connectome was not recognized prior to the development of a hyperaligned model of the human connectome.

## Beyond shared information: Individual differences in information encoded in fine-scale neural architecture

**Figure 10.**
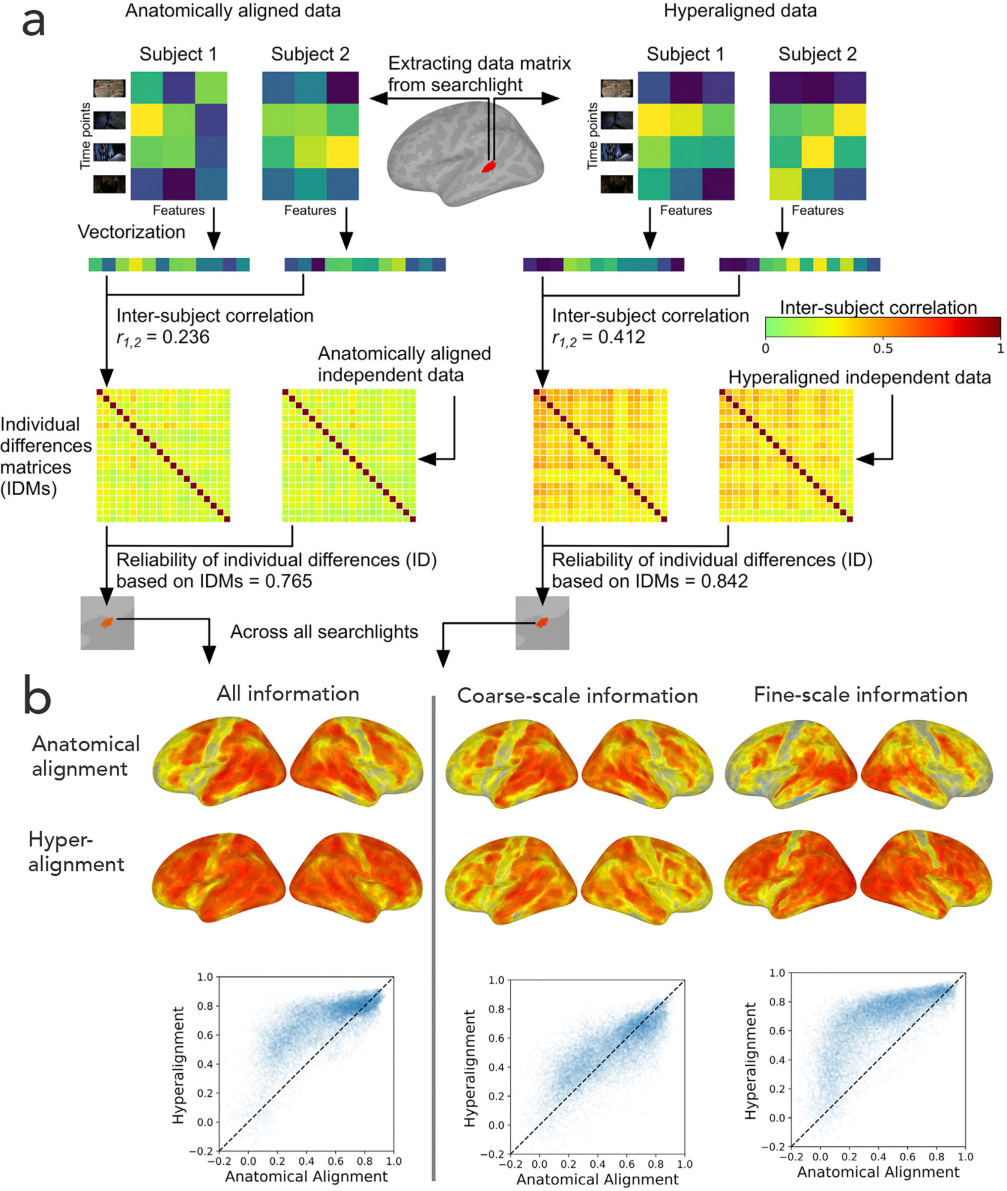
Reliability of individual differences in the information encoded in cortical topographies. (**a**) Response or functional connectivity data across vertices in a searchlight are vectorized for each individual, and the correlation between individuals is used as an index of similarity. Correlations between all pairs of individual brains are assembled in individual differences matrices (IDMs). Correlations between IDMs constructed from independent data sets are indices of the reliability of individual differences. After hyperalignment, individuals’ data are more similar, but the residual differences are more reliable than those calculated from anatomically aligned data, as shown for one searchlight in a and for 82% of searchlights as shown on the left in (**b**). The increase in reliability is due primarily to information encoded in fine-scale patterns, as shown on the right in b. Adapted from Figure 1 and 3, Feilong et al., 2018).

The goal of hyperalignment is to model the shared information encoded in idiosyncratic cortical topographies, but brains also differ in the information that they encode and process (Dubois and Adolphs, 2016). To investigate individual differences in encoded information, we calculated the intersubject correlations of the concatenated functional profiles for all vertices in a region for anatomically-aligned and hyperaligned data (Feilong et al., 2018; Figure 10a). From these pairwise comparisons we constructed a matrix of dissimilarities between all pairs of brains – an individual differences matrix (IDM). IDMs can be constructed based on response patterns or on functional connectivity patterns. Surprisingly, even though hyperalignment makes individual differences much smaller, as expected, the residual differences are substantially more reliable across independent data samples than those based on anatomically aligned data, and this increased reliability can be attributed to individual differences in the information encoded in fine-scale topographies (Figure 10b). The reliabilities of individual differences based on coarse-scale topographies are effectively equivalent for anatomically-aligned and hyperaligned data, whereas the individual differences based on residual data in fine-scale topographies are markedly more reliable for hyperaligned data than for anatomically-aligned data. The individual differences in the information encoded in fine-scale topographies also are more predictive of cognitive differences, such as general intelligence (Feilong et al., 2019). Previous work using anatomically-aligned connectomes has investigated only individual differences in coarse scale, inter-areal connectivity (Braga and Buckner, 2017; Dubois et al., 2018; Finn et al., 2015; Gordon et al., 2017; Kong et al., 2019). Restricting analysis to differences in coarse-scale connectivity is necessary with anatomically-aligned (or MSM-all aligned) data because differences in fine-scale connectivity are obscured by misalignment, but it appears that the most informative individual differences in cortical function reside in fine-scale patterns. Hyperalignment affords investigation of differences at this level of organization and factors out confounding variability due to topographic misalignment.

## Related methods for modeling shared information encoded in neural activity

Other approaches to modeling shared information in cortical topographies have mostly overlooked information encoded in fine-scale topographic patterns. Most have focused on areal structure (Gordon et al., 2017; Langs et al., 2016; Langs et al., 2014; Yeo et al., 2011) and describe the information in each area or network of connected areas in terms of a more global function, such as motor production, vision, attention, working memory, or theory of mind.

Other approaches decompose tuning profiles using analyses such as ICA, PCA, or factor analysis. These approaches have the potential to capture fine spatial scale variation in voxel-to-voxel differences in the mixture of components. Such variation, however, is often discarded because it is blurred in group analyses using anatomically aligned data (e.g. Filippini et al., 2009), or no attempt is made to model local topographies and the number of components is too limited to capture fine-scale structure (Just et al., 2010; Langs et al., 2016; Langs et al., 2014). Canonical Correlation Analysis (CCA) offers another method for finding shared information in two multivariate datasets (McIntosh and Mišić, 2013; Wang et al., 2020) but is not constrained to preserve vector geometry. Consequently, attempts to use CCA as a basis for hyperalignment have underperformed methods that do preserve vector geometry, unless CCA is regularized to make transformations mostly orthogonal (Xu et al., 2012).

Representational similarity analysis (RSA; Kriegeskorte et al., 2008a; Kriegeskorte and Kievit, 2013) and pattern component modeling (Diedrichsen and Kriegeskorte, 2017) are closely related to hyperalignment. These approaches focus on the vector geometry of response patterns in cortical areas as the common basis for representation of information that is shared across subjects and reveals individual differences (Charest et al., 2014). These approaches treat the topographies that embed these geometries as unimportant and, consequently, do not offer an explicit method for modeling the idiosyncrasies in local fine-scale topographies or a principled approach to rearrange information in the cortical sheet to enhance the inter-subject similarity of representational geometry in a cortical field.

The forward encoding approach models each voxel’s response profile independently as a weighted sum of stimulus features and, thereby, captures fine spatial-scale variation (Kay et al., 2008; Mitchell et al., 2008; Naselaris et al., 2011; Naselaris et al., 2009; Nishimoto et al., 2011). The great strength of this approach is that it attempts to define the information that is shared in terms of features, such as visual, acoustic, phonological, syntactic, and semantic features, thus specifying the content of shared information and affording prediction of responses to new stimuli if they can be modeled as a mixture of the features in the model. Hyperalignment and related methods afford improved cross-subject voxelwise prediction of response profiles (Bilenko and Gallant, 2016; Güçlü and van Gerven, 2017; Nastase et al., 2020; Van Uden et al., 2018). Forward encoding predefines the shared information by the stimulus feature model, and its validity is dependent on the validity and strength of that feature model. It does not provide any principled way to discover the structure of information that is encoded in neural responses that is not captured by the predefined feature model. These approaches also do not attempt to investigate neural responses as population codes with a representational geometry, but simply as independent voxel-wise responses to stimulus features. Voxel-wise response mapping studies tend to describe the topographies that result as gradients (Huth et al., 2016; Huth et al., 2012) with no attempt to model other aspects of fine-scale structure or their idiosyncratic variation across brains. The information space is typically reduced to a small number of dimensions (four in Huth et al., 2012).

## Discussion

The conceptual framework that we review uses hyperalignment to model cortical function by aligning the information represented in population responses in a common, high-dimensional information space. This framework does not align the cortical topographic patterns in a canonical anatomical topography. Information is defined computationally as the distances between population response vectors, which index reliable distinctions and similarities. By aligning response vectors from different subjects’ idiosyncratic topographies into a single information space with shared coordinate axes, information – the distinctions and similarities among response vectors – is now supported by the same basis functions across brains.

This computational framework can also be applied to functional connectivity data, replacing population response pattern vectors with topographic patterns of connectivities to a target, essentially a population connectivity vector. By aligning population connectivity vectors in a common information space, the connectivity profiles for each dimension in this space are markedly more similar across brains than are connectivity profiles for anatomically-aligned cortical loci. Not surprisingly, transformation matrices based on connectivity hyperalignment also align response pattern vectors, affording improved bsMVPC and higher ISCs of representational geometry.

Hyperalignment also models the idiosyncratic topographies in individual brains that carry shared information. Using the weights in individual transformation matrices for cortical loci as basis functions, a pattern vector in the common model space predicts a different topographic pattern in each brain. Boundaries for functional regions can be estimated in individual brains with high fidelity based on other subjects’ data, but the one-dimensional linear discriminants that define these regions account for only a small portion of the variance in cortical responses to naturalistic stimuli, indicating that they play a minor role. Functional topographies are modeled more comprehensively by our model, which posits a high-dimensional set of overlapping topographic basis functions.

### Rebalancing the roles of information content in neural populations and the functional profiles of individual units

The conceptual framework for hyperalignment is predicated on the hypothesis that information content that is shared across brains is instantiated in varied, idiosyncratic population codes. Hyperalignment models shared information by preserving the interrelations among pattern vectors – vector geometry – in a common information space while remixing the functional profiles of constituent dimensions – response tuning and functional connectivity profiles – thereby reshaping the functional profiles of the units in cortical patterns. Thus, the functional profiles of individual units are modeled as secondary to the information content that they support as populations. The commonality across brains is not modeled as common functional profiles of the individual units or as a common spatial distribution of functional profiles.

Traditionally, neuroscientists have focused on the tuning and connectivity profiles of individual units – neurons or voxels – and the information in population responses is modeled as a secondary consequence of the aggregation of those profiles (e.g. Gross et al., 1972; Hubel and Wiesel, 1959; Huth et al., 2016; Huth et al., 2012; Kay et al., 2008; Kiani et al., 2007; Sheinberg and Logothetis, 2001). The preservation of information content in vector geometry over variations in the functional profiles of individual units, however, suggests that the unit profiles are shaped to support the geometry, not that the vector geometry is merely a reflection of unit profiles.

Understanding how unit profiles can be shaped to support the requisite (and shared) population information is a matter that requires further theoretical work and experimental validation. Unit profiles clearly develop with experience and show a perplexing complexity and variety when considered in isolation (Çukur et al., 2013; Kiani et al., 2007; Sheinberg and Logothetis, 2001), especially responses and connectivity in studies with naturalistic stimuli (Çukur et al., 2013; McMahon et al., 2015; Park et al., 2017). Tuning functions of varying levels of coarseness – responses to related stimuli – support similarity of population responses and connectivity patterns (Kiani et al., 2007) and, consequently, pattern vector geometries. Relationships reflected in similarity of responses can be due to shared perceptual features or cognitive factors such as agency (Connolly et al., 2012; Sha et al., 2015; Thorat et al., 2019) and action goals (Nastase et al., 2017). The relationships reflected in representational geometry differ by cortical field (Borghesani et al., 2016; Freiwald and Tsao, 2010; Guntupalli et al., 2017; Guntupalli et al., 2016; Visconti di Oleggio Castello et al., 2017), and these differences reflect processing or the disentangling of target information from confounds (DiCarlo et al., 2012; DiCarlo and Cox, 2007; Kriegeskorte and Kievit, 2013). The topographic organization of units with particular functional profiles may arise over the course of development and with varying experience (Arcaro et al., 2017). These functional profiles may in fact fluctuate over time despite retaining the information encoded at the population level (Gallego et al., 2020). The functional profiles of individual units need not be easily-interpretable (Ponce et al., 2019). Differences in vector geometry across cortical fields in a processing system are supported by different configurations of tuning profiles of individual units. The composition of varied response profiles in populations, therefore, is shaped to create the varied information spaces that are required in a processing system, a learning process that may rest on feedback among the cortical fields in the system. Deep convolutional neural networks (CNNs) provide a computational instantiation of a learning process guided by feedback that produces varied sets of individual units that perform the same functions. Like cortical systems, the individual features of CNNs vary based on initialization and network architecture, but the similarity structure of feature vectors reveals commonality of layer-specific information content across these variations (Kornblith et al., 2019).

### Cortical functional architecture is a degenerate system

The contrast between a common deep structure for information that is instantiated in variable surface structure has a parallel in language (Chomsky, 1965; Hockett, 1958). The same meaning can be conveyed linguistically with substantial variation in wording and sentence structure, including the words and syntax of different languages. Analogous to the variable surface structure of language and the deep structure of meaning in linguistic communication, the variable surface structure of cortical topographies affords representation of shared deep information structure.

More broadly, the conceptual framework of hyperalignment models a property of cortical functional architecture that is a ubiquitous property of complex biological systems, namely ‘degeneracy’ – ‘the ability of elements that are structurally different to perform the same function or yield the same output’ (Edelman and Gally, 2001). Degeneracy is a property of systems that evolve or develop or learn and are characterized by complex interactions among constituent elements. By contrast, designed systems usually try to avoid unplanned interactions. Unplanned interactions in degenerate systems allow unexpected new functions to emerge or to be learned. Different instantiations of elements that perform the same or similar functions may be differentially adaptive to changing demands, providing a basis for evolution. Degeneracy is characteristic of systems at all levels of biological organization from genetics to body movements and language. Hyperalignment models how the variable structural elements of functional cortical architecture – local neural responses and point-to-point functional connectivities – perform similar functions in aggregate for representation and processing of information and provides a basis for studying variations in instantiations of these functions, which are the basis for diversity in cognitive abilities.
